# Non-Linear Dynamic Analysis on Hybrid Air Bearing-Rotor System under Ultra-High Speed Condition

**DOI:** 10.3390/ma15020675

**Published:** 2022-01-17

**Authors:** Laiyun Song, Guoqin Yuan, Hongwen Zhang, Yalin Ding, Kai Cheng

**Affiliations:** Key Laboratory of Airborne Optical Imaging and Measurement, Changchun Institute of Optics, Fine Mechanics and Physics, Chinese Academy of Sciences, Changchun 130033, China; timsong1029@163.com (L.S.); zhang_hong_wen@sina.com (H.Z.); dingyl_1964@126.com (Y.D.); kai.cheng@brunel.ac.uk (K.C.)

**Keywords:** hybrid air spindle, nonlinear behavior, unbalanced mass, ultra-high speed, structure parameters

## Abstract

The non-linear dynamic behavior of a hybrid air bearing-rotor system is very complicated and requires careful attention when designing to avoid spindle failure, especially under ultra-high speed condition. In this paper, the rotor trajectory of a hybrid air bearing-rotor system is obtained by solving the unsteady Reynolds equation and motion equations simultaneously. The typical non-linear behavior of hybrid air bearing-rotor systems is illustrated with the analysis of the rotor trajectory, the phase angle, time domain vibration and power spectral density. Furthermore, the influences of the rotor mass, external load, rotating speed and unbalanced mass on the non-linear behavior are investigated. Finally, the effect of structure parameters on the rotor trajectory is studied and the phenomenon under ultra-high speed condition is illustrated, which provides some new guidelines on the ultra-high speed air spindle design.

## 1. Introduction

The production of optical lenses with high surface quality is mainly carried out on machines equipped with high-precision air spindles [1], due to their inherent advantages in high motion precision [2,3]. The high-precision air spindles operated at ultra-high speed condition (above 100,000 r/min) can be characterized as the ultra-high speed hybrid air spindle, in which the aerostatic effect and the aerodynamic effect are equivalent in the load capacity of the spindle [4]. The hybrid air bearing-rotor system is subjected to the combined effects of the non-linear force of the air bearings, the rotational inertia, the rotor gravity and the centrifugal force caused by the unbalanced mass of the rotor, resulting in a very complicated rotor trajectory [5,6]. Thus, the non-linear behavior and the rotor trajectory of the ultra-high speed hybrid air spindles need to be analyzed systematically under different working conditions and different structures, so as to provide theoretical guidance for improving the rotation precision of the hybrid air spindle.

Therefore, many studies have simulated the rotor trajectory and analyzed its nonlinear rotor dynamics. Wang et al. adopted the hybrid analysis method of the bearing-rotor system to simulate the trajectory of the rotor, and accordingly analyzed the dynamic behavior of the high-speed aerostatic bearing-rotor system, such as periodic, sub-harmonic and quasi-periodic harmonic responses [6]. The hybrid method of solving the unsteady Reynold equation and the motion equations mainly combines a differential transformation method (DTM) and finite difference method (FDM). Furthermore, Wang et al. designed and calculated a series of non-linear dynamic parameters such as phase angle trajectory, power spectrum and bifurcation diagram [7] to study the non-linear behavior of the aerostatic bearings [8], porous bearings [9], self-acting bearings [10], etc., Belforte et al. designed a high-speed electrospindle for 100 krpm and analyzed the static and dynamic runout of the spindle by solving the time-domain Reynolds equation for gas film together with the equation of motion of the rotor [11]. Zhang studied the nonlinear behavior of the aerostatic bearing-rotor systems based on the bifurcation diagram, orbit of rotor center and frequency spectrum diagram [12]. They indicated that the increase of the supply pressure could restrict the non-linear behaviors. Kuo et al. investigated the non-linear behavior of the porous gas bearing systems by the method of bifurcation diagram and maximum Lyapunov exponents (MLE) [13]. High-precision machine learning was proposed to solve the MLE prediction efficiently. Li et al. established a five-degrees-of-freedom hybrid spindle model, which considered the influence of the tilt motion of the spindle on the non-linear behaviors, and further analyzed the dynamic response of the spindle system [14,15]. Guo et al. established an integrated non-linear dynamic model considering shaft motion, unsteady gas film and deformation of foil structure, which was proved by designed experiments [16].

The above references hardly mentioned the effect of unbalanced mass on the non-linear behavior of the hybrid air bearing, which is significant in the rotor-bearing system [17,18]. Du et al. used the combination of FEM and FDM methods to analyze the non-linear dynamic behavior of the semicircular bearing-rotor system with spiral grooves. Through the power spectrum and bifurcation diagram, the influence of the unbalanced mass of the spindle on the dynamic behavior of the system at different speeds was identified [19]. Guo et al. investigated the effect of unbalanced load on the non-linear response of the gas foil bearing-rotor systems, in which the frequency of the peak response increased with the increment of the unbalance load [20]. However, the rotating speed in their research was limited within 40 krpm, and the influences on the non-linear behavior of gas bearing-rotor system under ultra-high speed condition were still not clear.

In this paper, the non-linear behavior of hybrid gas journal bearings under ultra-high speed condition is studied by solving the unsteady Reynolds equation and rotor motion equations simultaneously. The typical non-linear behavior is illustrated and studied by means of the rotor trajectory, the phase angle, time domain vibration and power spectral density. Furthermore, the influences on the non-linear behavior of the hybrid air bearing-rotor systems are investigated considering the rotor mass, external load, rotating speed and unbalanced mass. Finally, the effect of the structure parameters on the rotor trajectory of a hybrid air bearing-rotor system under ultra-high rotating speed condition are studied and the effect of the ultra-high speed condition is emphasized, providing new guidelines for designing the ultra-high speed air spindle.

## 2. Mathematical Model

**Figure 1:**
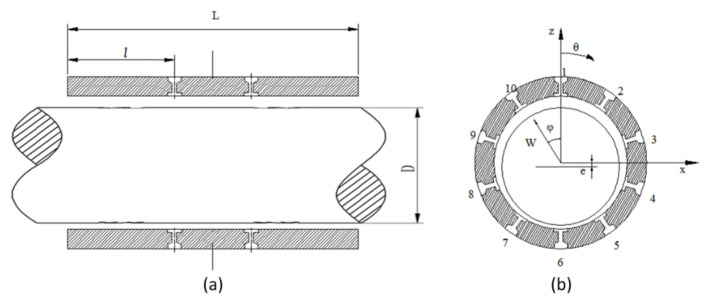
Schematic diagram of hybrid air bearings (**a**) axial direction, (**b**) circumferential direction.

The schematic diagram of the hybrid air spindle is shown in Figure 1. In order to calculate the center trajectory of the hybrid air spindle under ultra-high speed condition, the unsteady Reynolds equation needs to be solved to obtain the unsteady pressure distribution. The non-steady state Reynolds equation [21] is:

(1) $$\frac{\partial }{\partial \varsigma }\left(ph^{3}\frac{\partial p}{\partial \varsigma }\right)+\frac{\partial }{\partial z}\left(ph^{3}\frac{\partial p}{\partial z}\right)=6\eta u\frac{\partial (ph)}{\partial x}+12\eta \frac{\partial (ph)}{\partial t}$$

By employing the dimensionless parameters:

(2) $$P=p/p_{a},H=h/c,X=\xi /R,Z=z/L,\tau =tws,\lambda =w_{s}/w,\Lambda =\frac{6\eta wR^{2}}{p^{a}c^{2}}$$

The dimensionless non-steady state Reynolds equation is:

(3) $$\frac{\partial }{\partial X}\left(PH^{3}\frac{\partial P}{\partial X}\right)+\frac{\partial }{\partial Z}\left(PH^{3}\frac{\partial P}{\partial Z}\right)=\Lambda \frac{\partial (PH)}{\partial X}+2\Lambda \lambda \frac{\partial (PH)}{\partial \tau }$$

The equation is discretized using the FDM method:

(4) $$\begin{array}{l} \frac{\partial }{\partial X}\left(H^{3}\frac{\partial P^{2}}{\partial X}\right)=\frac{{\left(H^{3}\frac{\partial P^{2}}{\partial X}\right)}_{i,j+1/2}-{\left(H^{3}\frac{\partial P^{2}}{\partial X}\right)}_{i,j-1/2}}{\Delta X}\\ =H_{i,j+1/2}^{3}\frac{P_{i,j+1}^{2}-P_{i,j}^{2}}{{\left(\Delta X\right)}^{2}}-H_{i,j-1/2}^{3}\frac{P_{i,j}^{2}-P_{i,j-1}^{2}}{{\left(\Delta X\right)}^{2}} \end{array}$$

(5) $$\begin{array}{l} \frac{\partial }{\partial Z}\left(PH^{3}\frac{\partial P^{2}}{\partial Z}\right)=\frac{{\left(H^{3}\frac{\partial P^{2}}{\partial Z}\right)}_{i+1/2,j}-{\left(H^{3}\frac{\partial P^{2}}{\partial Z}\right)}_{i-1/2,j}}{\Delta Z}\\ =H_{i+1/2,j}^{3}\frac{P_{i+1,j}^{2}-P_{i,j}^{2}}{{\left(\Delta Z\right)}^{2}}-H_{i-1/2,j}^{3}\frac{P_{i,j}^{2}-P_{i-1,j}^{2}}{{\left(\Delta Z\right)}^{2}} \end{array}$$

(6) $$2\Lambda \frac{\partial (PH)}{\partial X}=\Lambda \frac{\left(P_{i,j+1}H_{i,j+1}-P_{i,j-1}H_{i,j-1}\right)}{\Delta X}$$

(7) $$2\Lambda \lambda \frac{\partial (PH)}{\partial \tau }=\frac{2\Lambda }{w}[H_{i,j}^{n}\frac{P_{i,j}^{n+1}-P_{i,j}^{n}}{\Delta t}+P_{i,j}^{n}\frac{H_{i,j}^{n+1}-H_{i,j}^{n}}{\Delta t}]$$

where $P_{i,j}^{n}$ is the pressure at the point (*i*, *j*) of the current time step while $P_{i,j}^{n+1}$ is the pressure at the point (*i*, *j*) of the next time step. $H_{i,j}^{n}$ is the film thickness at the point (*i*, *j*) of the current time step while $H_{i,j}^{n+1}$ is the film thickness at the point (*i*, *j*) of the next time step.

Therefore, the pressure distribution of the next time step can be calculated from the pressure distribution of the previous time step by formulas (4)–(7):

(8) $$\begin{array}{l} P_{i,j}^{n+1}=A_{i,j}P_{i,j+1}^{n}{}^{2}+B_{i,j}P_{i,j-1}^{n}{}^{2}+C_{i,j}P_{i+1,j}^{n}{}^{2}+D_{i,j}P_{i-1,j}^{n}{}^{2}+E_{i,j}P_{i,j}^{n}{}^{2}\\ +F_{i,j}P_{i,j+1}^{n}+G_{i,j}P_{i,j-1}^{n}+K_{i,j}P_{i,j}^{n} \end{array}$$

where

(9) $$\begin{array}{l} A_{i,j}=-\frac{w\Delta t}{2\Lambda H_{i,j}^{n}}\frac{H_{i,j+1/2}^{n}{}^{3}}{\Delta X^{2}}\\ B_{i,j}=\frac{w\Delta t}{2\Lambda H_{i,j}^{n}}\frac{H_{i,j-1/2}^{n}{}^{3}}{\Delta X^{2}}\\ C_{i,j}=-\frac{w\Delta t}{2\Lambda H_{i,j}^{n}}\frac{H_{i+1/2,j}^{n}{}^{3}}{\Delta Z^{2}}\\ D_{i,j}=-\frac{w\Delta t}{2\Lambda H_{i,j}^{n}}\frac{H_{i-1/2,j}^{n}{}^{3}}{\Delta Z^{2}}\\ E_{i,j}=-\frac{w\Delta t}{2\Lambda H_{i,j}^{n}}(\frac{H_{i,j+1/2}^{n}{}^{3}}{\Delta X^{2}}+\frac{H_{i,j-1/2}^{n}{}^{3}}{\Delta X^{2}}+\frac{H_{i+1/2,j}^{n}{}^{3}}{\Delta Z^{2}}+\frac{H_{i-1/2,j}^{n}{}^{3}}{\Delta Z^{2}})\\ F_{i,j}=-\frac{w\Delta t}{2H_{i,j}^{n}}\frac{H_{i,j+1}^{n}}{\Delta X}\\ G_{i,j}=\frac{w\Delta t}{2H_{i,j}^{n}}\frac{H_{i,j-1}^{n}}{\Delta X}\\ K_{i,j}=(1-\frac{H_{i,j}^{n+1}-H_{i,j}^{n}}{H_{i,j}^{n}}) \end{array}$$

The boundary condition in the FDM is as below:

(1) atmosphere boundary condition: $P(Z=0)=p_{a}/p_{s}$, $P(Z=1)=p_{a}/p_{s}$.
(2) symmetric boundary condition: P(X=0)=P(X=2π).
(3) air film pressure at orifice: $P(orifice\_position)=p_{d}/p_{s}$.

where *p_d_* is the downstream pressure at the feeder. As Figure 1 depicts, the pressure is reduced from *p_s_* to *p_d_* through the feeder. Due to the existence of the recess, there is always π*d*^2^/4 < π*d_r_h*, where *d_r_* is the diameter of the recess. As a result, the feeder is a typical orifice [22], and its flow rate is derived as:

(10) $$\overset{\cdot }{m_{t}}=\frac{\pi d^{2}}{4}p_{s}\sqrt{\frac{2\rho_{a}}{p_{a}}}\psi_{s}$$

(11) $$\psi_{s}=\left\{ \begin{array}{l} {\left[\frac{\kappa }{2}{\left(\frac{2}{\kappa +1}\right)}^{\left(\kappa +1)/(\kappa -1\right)}\right]}^{1/2};(\frac{p_{d}}{p_{s}}\leq \beta_{\kappa })\\ \frac{\kappa }{\kappa -1}[{\left(\frac{p_{d}}{p_{s}}\right)}^{\kappa /2}-{\left(\frac{p_{d}}{p_{s}}\right)}^{(\kappa +1)/\kappa }];(\frac{p_{d}}{p_{s}}\geq \beta_{\kappa }) \end{array}\right.$$

To solve the *p_d_* the mass flow rate equation is solved simultaneously with the unsteady Reynold equation.

To calculate the unsteady-state Reynolds equation, it is necessary to discretize in time steps. The Reynolds equation is solved at each time step, and the pressure distribution and gas film thickness is updated according to the motion equations. Note that the time step should be short enough to meet the convergence criterion [11], thus *dt* = 10^−6^ s in this paper.

First, the initial conditions are provided: the initial *P*_0_ and *H*_0_ (pressure and gas film thickness at time *t* = 0) is calculated by the steady Reynolds equation. Then the *x*-direction and *y*-direction forces are obtained based on the pressure distribution. Finally, the forces are converted into acceleration, velocity, and displacement.

The initial conditions are:

(12) $$\begin{array}{l} x_{Gq}=0\\ y_{Gq}=0.5e^{-6}\\ \overset{•}{x_{Gq}}=0\\ \overset{•}{y_{Gq}}=0\\ e_{Gq}=0 \end{array}$$

According to the initial conditions, the initial bearing eccentricity is substituted into the steady-state Reynolds equation, therefore *P*_0_ and *H*_0_ are obtained. Then *F_X_* and *F_Y_* can be deduced as:

(13) $$\left(\begin{array}{l} F_{x}\\ F_{y} \end{array}\right)=p_{0}RL\int_{0}^{1}\int_{0}^{2\pi }P_{0}\left(\begin{array}{l} \sin X\\ -\cos X \end{array}\right)dXdZ$$

Furthermore, according to Newton’s second law and kinematic relationship:

(14) $$\begin{array}{l} m\overset{••}{x_{G}}=F_{x}+m\varepsilon w^{2}\cos wt+m_{u}\rho_{r}\overset{•}{w}\cos wt\\ \overset{••}{my_{G}}=F_{y}+m\varepsilon w^{2}\sin wt+m_{u}\rho_{r}\overset{•}{w}\cos wt\\ \overset{•}{x_{G}}=\overset{•}{x_{Gq}+}\overset{••}{x_{G}}\Delta t\\ \overset{•}{y_{G}}=\overset{•}{y_{Gq}}+\overset{•}{y_{G}}\Delta t\\ x_{G}=x_{Gq}+\overset{•}{x_{G}}\Delta t+\frac{1}{2}\overset{••}{x_{G}}\Delta t\\ y_{G}=y_{Gq}+\overset{•}{y_{G}}\Delta t+\frac{1}{2}\overset{••}{y_{G}}\Delta t\\ e_{G}=\sqrt{x_{G}{}^{2}+y_{G}{}^{2}}\\ \tan\theta =\frac{y_{G}}{x_{G}} \end{array}$$

where *m* is the rotor mass, *m_u_* is the unbalanced mass, *ρ_r_* is the unbalanced mass vector, *x_Gq_* and *y_Gq_* represent the initial position in the *x*- and *y*-direction.

**Figure 2:**
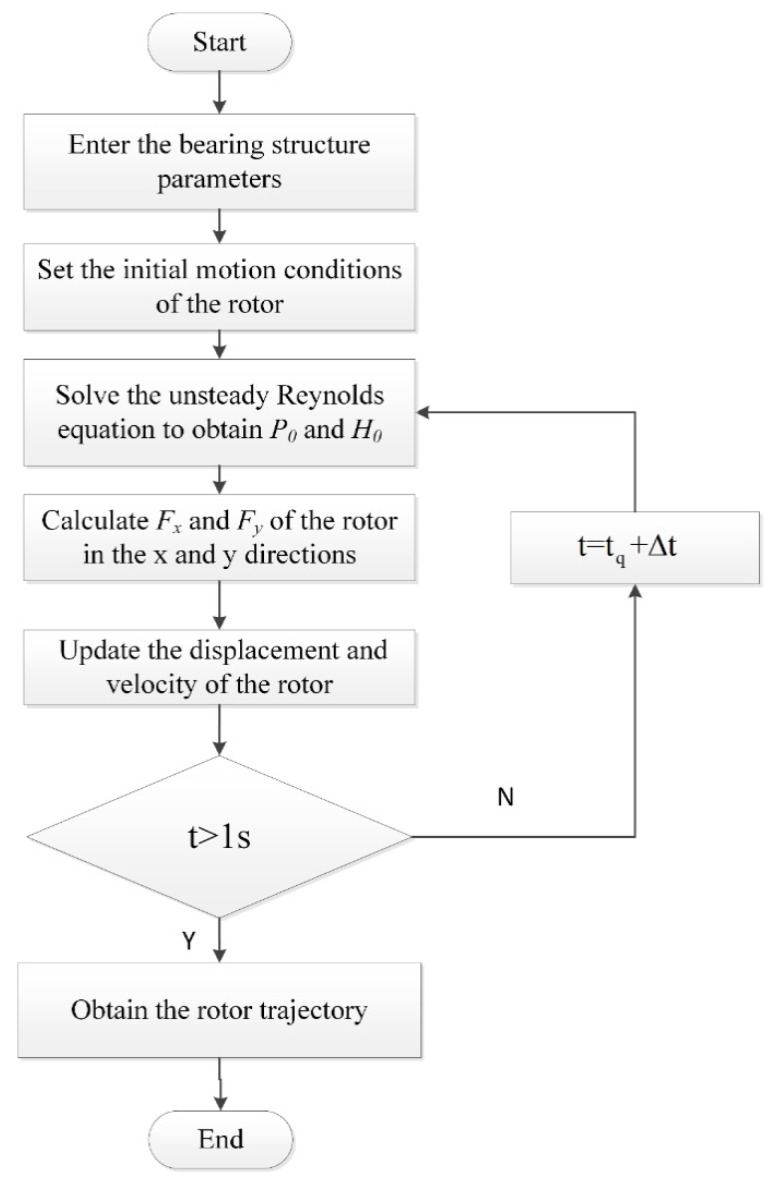
Flow chart of rotor trajectory calculation.

Then the pressure of the next time step is calculated by substituting the new bearing eccentricity, attitude angle, the gas film thickness and pressure distribution of the previous step into formulas (4)–(9). The calculation process is shown in Figure 2. The unsteady pressure distribution and the spindle center in the time domain are obtained by iterating the process, and the structural parameters of the bearing are shown in Table 1.

## 3. Results and Discussion

**Figure 3:**
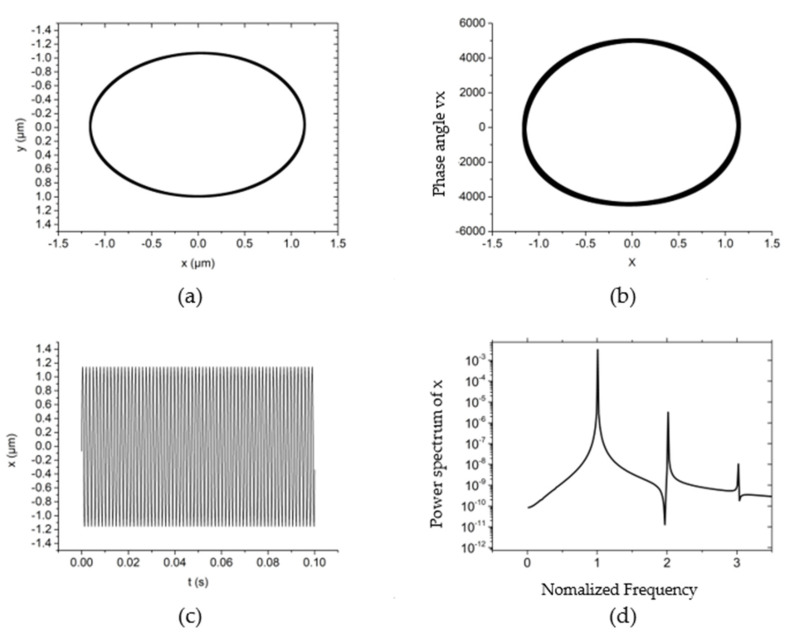
The non-linear dynamic behavior of the rotor at *w* = 30,000 r/min, *Fy* = 10 N, *m_u_* = 0.01 g, *m* = 30 g. (**a**) rotor center trajectory, (**b**) phase angle in *x*-direction, (**c**) x displacement time-domain curve, (**d**) power spectral density in the *x*-direction.

**Figure 4:**
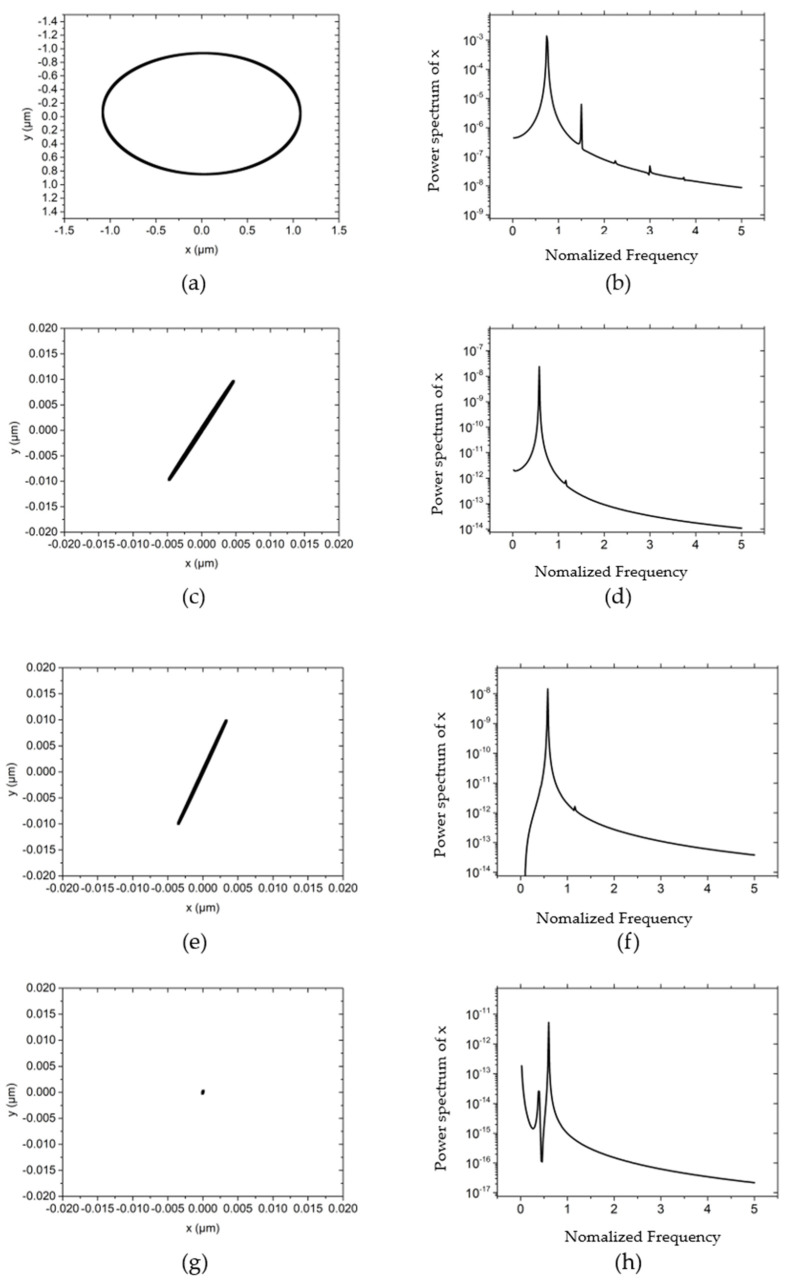
The influence of different rotor mass on rotor trajectory and power spectral density *w* = 50,000 r/min, *Fy* = 10 N, *m_u_* = 0.01 g. (**a**) Rotor center trajectory (*m* = 50 g), (**b**) Power spectral density in the *x* direction (*m* = 50 g), (**c**) Rotor center trajectory (*m* = 90 g), (**d**) Power spectral density in the *x* direction (*m* = 90 g), (**e**) Rotor center trajectory (*m* = 120 g), (**f**) Power spectral density in the *x* direction (*m* = 120 g), (**g**) Rotor center trajectory (*m* = 150 g), (**h**) Power spectral density in the *x* direction (*m* = 150 g).

**Figure 5:**
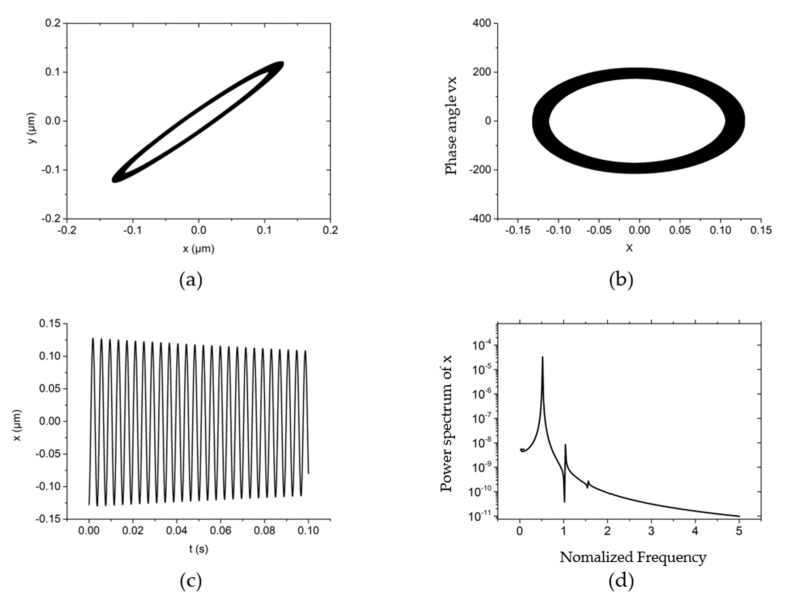
The nonlinear dynamic behavior of the rotor at *w* = 50,000 r/min, *Fy* = 10 N, *m_u_* = 0.01 g, *m* = 90 g. (**a**) Rotor center trajectory, (**b**) Phase angle in *x* direction, (**c**) X displacement time-domain curve, (**d**) Power spectral density in the *x* direction.

**Figure 6:**
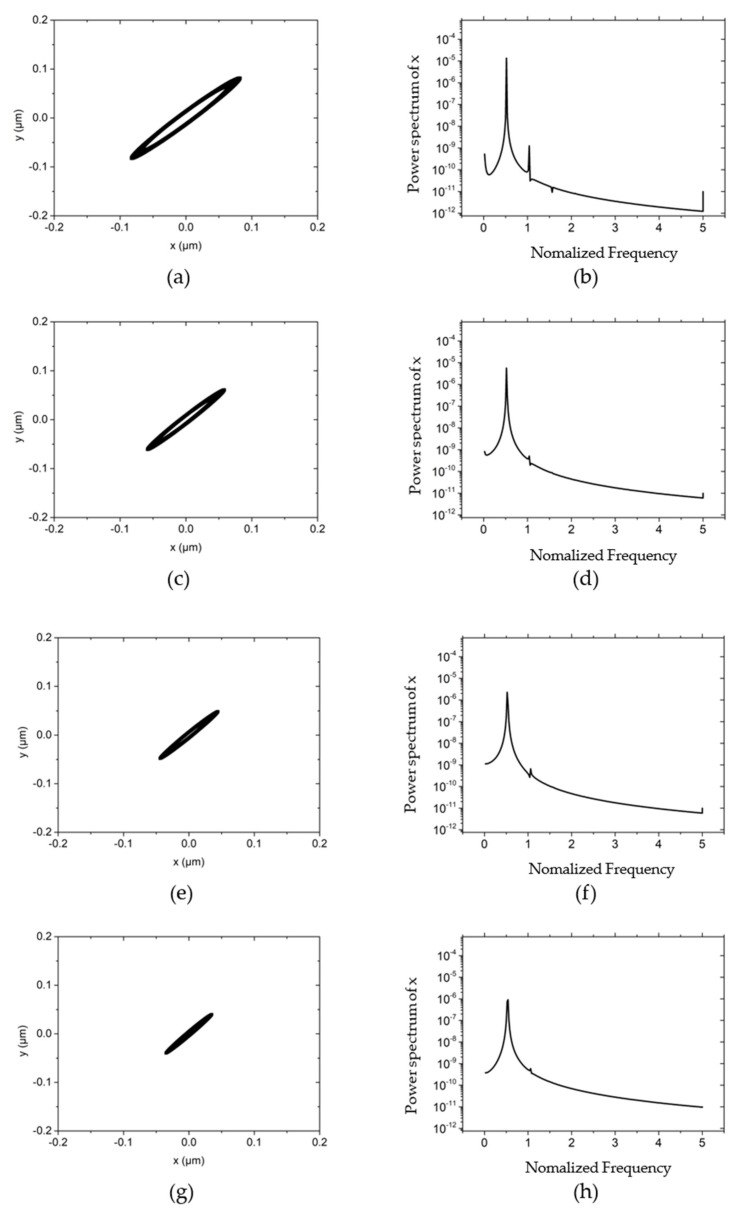
The influence of different rotor mass on rotor trajectory and power spectral density at *w* = 50,000 r/min, *m_u_* = 0.01 g, *m* = 90 g. (**a**) Rotor center trajectory (*Fy* = 20 N), (**b**) Power spectral density in the *x* direction (*Fy* = 20 N), (**c**) Rotor center trajectory (*Fy* = 30N), (**d**) Power spectral density in the *x* direction (*Fy* = 30 N), (**e**) Rotor center trajectory (*Fy* = 40 N), (**f**) Power spectral density in the *x* direction (*Fy* = 40 N), (**g**) Rotor center trajectory (*Fy* = 50 N), (**h**) Power spectral density in the *x* direction (*Fy* = 50 N).

**Figure 7:**
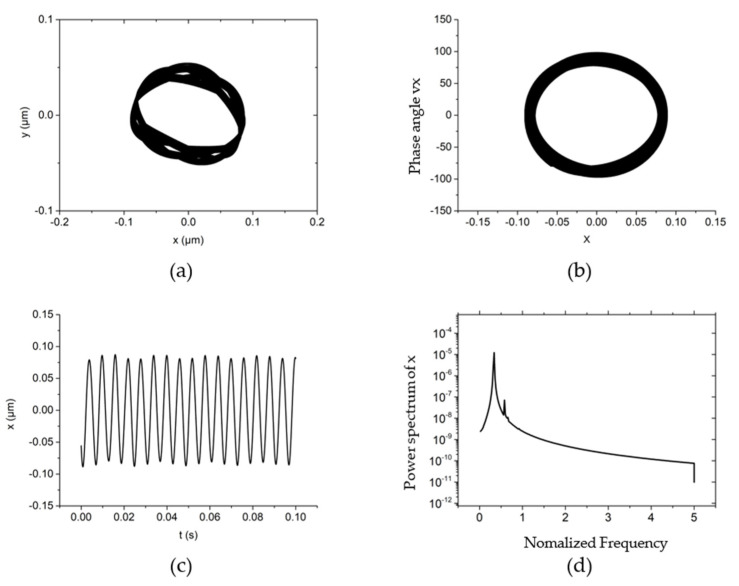
The non−linear dynamic behavior of the rotor at *w* = 50,000 r/min, *Fy* = 30 N, *m_u_* = 0.1 g, *m* = 90 g. (**a**) Rotor center trajectory, (**b**) Phase angle in *x* direction, (**c**) X displacement time-domain curve, (**d**) Power spectral density in the *x* direction.

**Figure 8:**
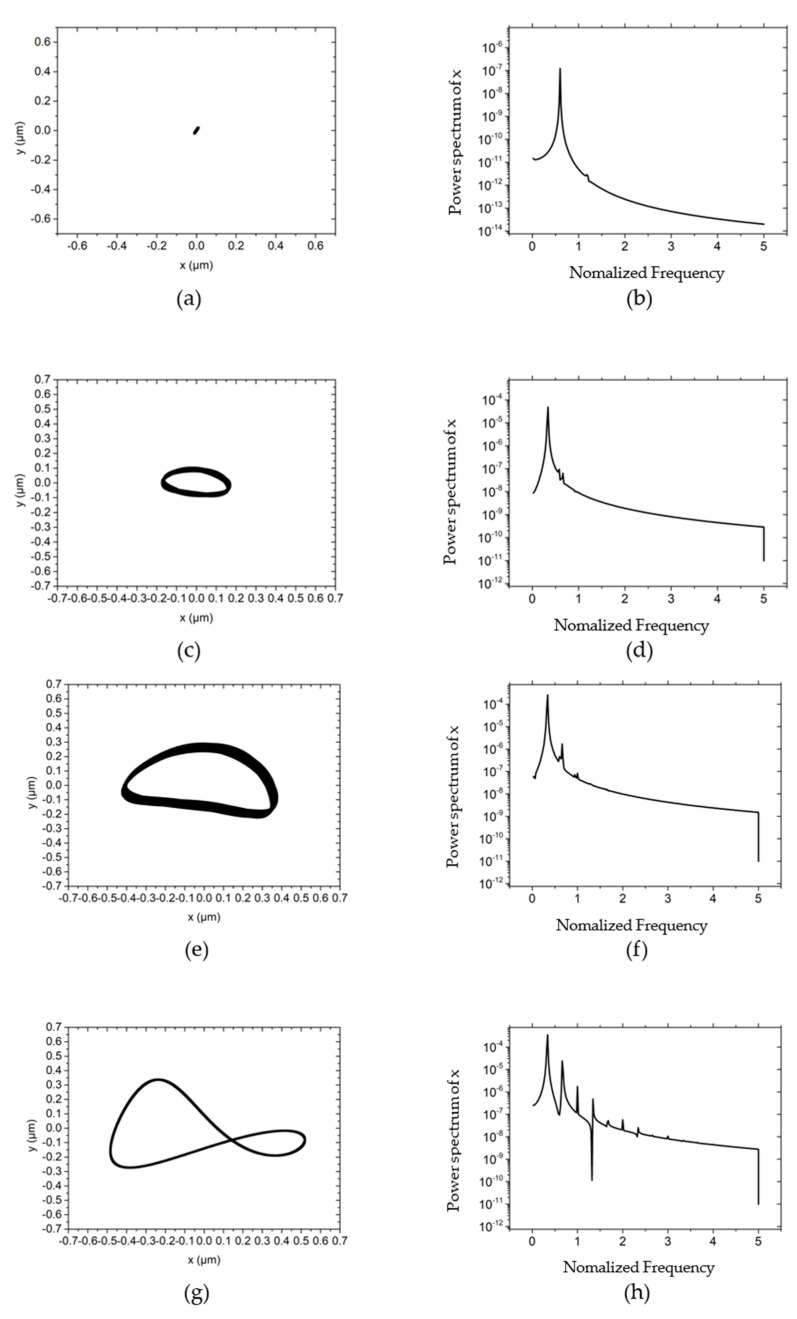

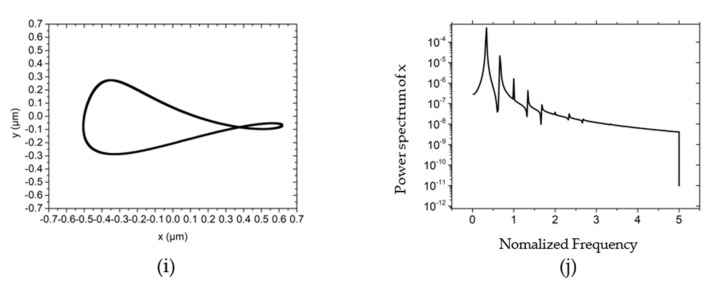
The influence of different rotor mass on rotor trajectory and power spectral density at *w* = 50,000 r/min, *Fy* = 30 N, *m_u_* = 0.1 g, *m* = 90 g. (**a**) Rotor center trajectory (*m_u_* = 0), (**b**) Power spectral density in the *x* direction (*m_u_* = 0), (**c**) Rotor center trajectory (*m_u_* = 0.2), (**d**) Power spectral density in the *x* direction (*m_u_* = 0.2), (**e**) Rotor center trajectory (*m_u_* = 0.3), (**f**) Power spectral density in the *x* direction (*m_u_* = 0.3), (**g**) Rotor center trajectory (*m_u_* = 0.4), (**h**) Power spectral density in the *x* direction (*m_u_* = 0.4), (**i**) Rotor center trajectory (*m_u_* = 0.5), (**j**) Power spectral density in the *x* direction (*m_u_* = 0.5).

**Figure 9:**
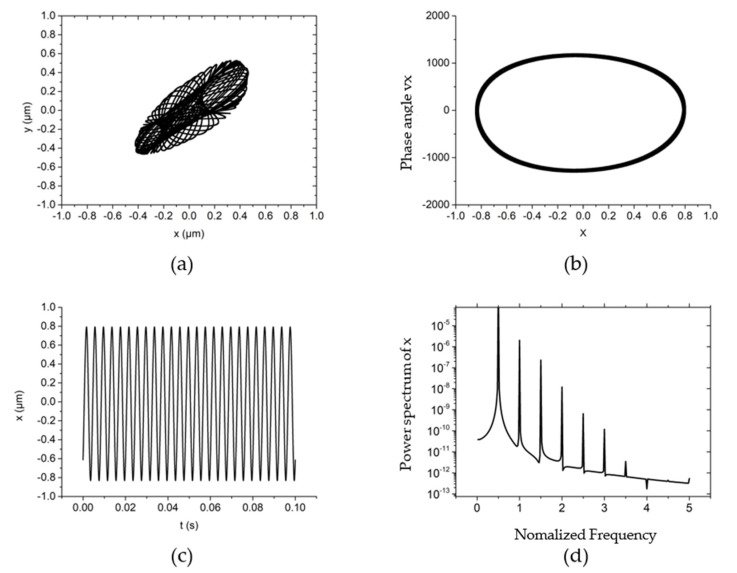
The non−linear dynamic behavior of the rotor at *w* = 50,000 r/min, *Fy* = 30 N, *m_u_* = 0.1 g, *m* = 90 g. (**a**) Rotor center trajectory, (**b**) Phase angle in *x* direction, (**c**) X displacement time-domain curve, (**d**) Power spectral density in the *x* direction.

**Figure 10:**
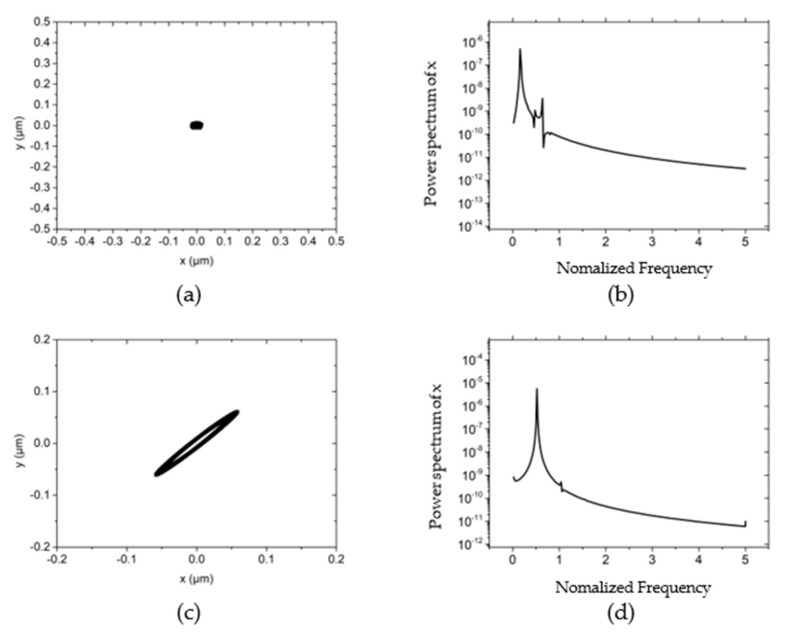

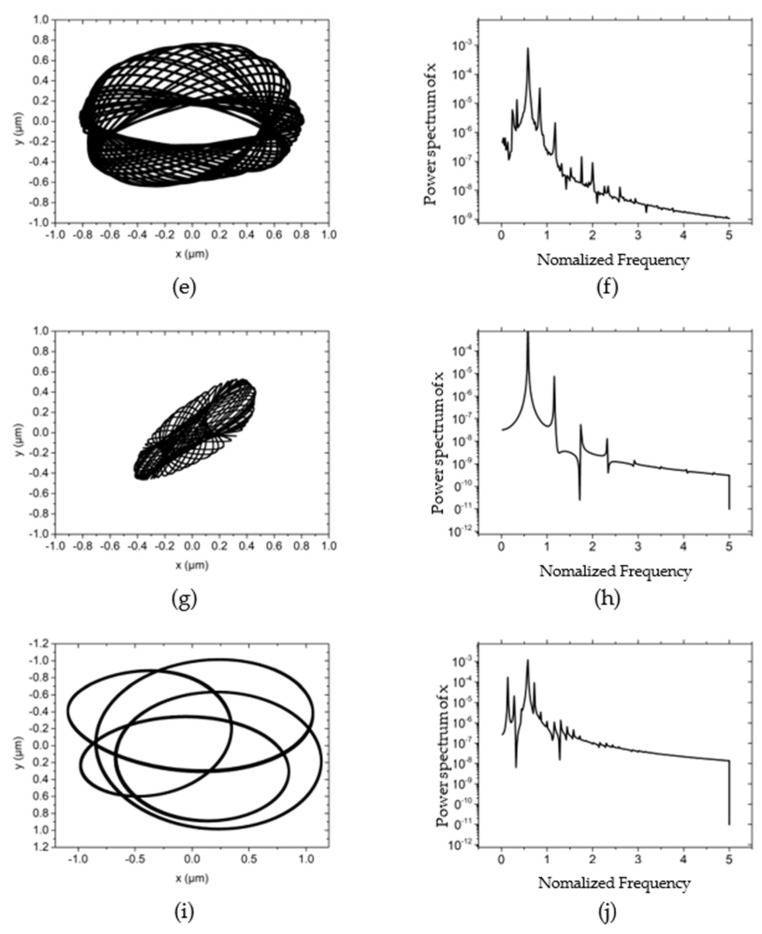
The influence of different rotor mass on rotor trajectory and power spectral density at *Fy* = 30 N, *m_u_* = 0.01 g, *m* = 90 g. (**a**) Rotor center trajectory (*w* = 10,000 r/min), (**b**) Power spectral density in the *x* direction (*w* = 10,000 r/min), (**c**) Rotor center trajectory (*w* = 30,000 r/min), (**d**) Power spectral density in the *x* direction (*w* = 30,000 r/min), (**e**) Rotor center trajectory (*w* = 40,000 r/min), (**f**) Power spectral density in the *x* direction (*w* = 40,000 r/min), (**g**) Rotor center trajectory (*w* = 90,000 r/min), (**h**) Power spectral density in the *x* direction (*w* = 90,000 r/min), (**i**) Rotor center trajectory (*w* = 140,000 r/min), (**j**) Power spectral density in the *x* direction (*w* = 140,000 r/min).

On the basis of the numerical model in Section 2, the typical non-linear dynamic behaviors of the hybrid air bearing-rotor system are analyzed in Figure 3, Figure 4, Figure 5, Figure 6, Figure 7, Figure 8, Figure 9 and Figure 10 in terms of rotor center trajectory, phase angle and time-domain vibration and power spectral density. Furthermore, the influences of the rotor mass, load force, rotating speed and unbalanced mass on the non-linear behavior of the hybrid air bearing-rotor system are studied and demonstrated using non-linear analysis.

The non-linear dynamic behavior of the system is shown in Figure 3 when the rotating speed is 30,000 r/min, the system external load (vertical direction) is 10 N, the rotor mass is 10 g and the unbalanced mass is 0.01 g. From Figure 3a,b, it can be seen that the trajectory and phase angle of the rotor are both an ellipse under this working condition. Meanwhile, the rotor is characterized as a period vibration, as shown in the time domain vibration diagram of Figure 3c,d. Figure 3d shows that under this kind of motion characteristics, the main vibration energy of the rotor comes from the rotation speed vibration of the rotor, and the peak energy density of the system is also near the rotation frequency. The vibration of the system at higher frequencies is also a multiple of the rotation frequency.

Figure 4 illustrates the influences of different rotor masses on the rotor center trajectory and power spectral density at the working condition that the rotating speed is 30,000 r/min, the external load of the system (vertical direction) is 10 N, and the unbalanced mass is 0.01 g. By comparing Figure 3a with Figure 4a,c,e, it can be concluded that as the mass of the rotor increases, the trajectory of the rotor changes from an ellipse with an amplitude of about 1 μm to a small ellipse with an amplitude of 0.01 μm. The long axis of the ellipse remains approximately 60° with the *x*-axis with the variation of the rotor mass. This is because as the mass increases, the natural frequency of the system decreases and the whirl frequency becomes smaller by consequence. According to the analysis in reference [21], the reduction of the whirl frequency increases the damping of the rotor system, thereby restricting the trajectory of the rotor to a very low level. By comparing the power spectral density in the *x*-direction, it can also be known: when the rotor mass is low, the power spectral density peak appears at about a single speed, and the peak value is 10^−3^~10^−4^. As the mass further increases, the phenomenon of “half speed whirl” appears, with a peak value of 10^−7^~10^−8^. The mass of the rotor is further increased to 150 g, and the damping of the hybrid air bearing-rotor system is further increased. Thus the rotation of the rotor gradually tends to a point, and the overall power spectral density is reduced to below 10^−11^, as shown in Figure 4g,h.

One of the typical non-linear dynamic behaviors of the system is shown in Figure 5 when the rotating speed is 50,000 r/min, the system external load (vertical direction) is 10 N, the rotor mass is 90 g and the unbalanced mass is 0.01 g. Under this rotating speed, the trajectory of the rotor is an ellipse with an amplitude of about 0.2 μm, and the direction of the polar axis is the same as that in Figure 4c. Due to the slight increase in rotating speed, the longitudinal polar axis is larger, and the elliptical trajectory in the figure is more obvious. The phase angle under this system is a belt-type ellipse, which is due to the phase angle vibration caused by the unbalanced mass, as shown in Figure 5b. Figure 5d clearly shows that under the typical non-linear dynamic behavior, the half-speed whirl motion begins to appear. This is because the energy to maintain the rotational motion caused by the fluid comes from the rotor movement, and the influences is enhanced with the rotating speed increases.

When the rotating speed is 50,000 r/min, the rotor mass is 90 g and the unbalanced mass is 0.01 g, the influence of different external loads on the non-linear dynamics of the hybrid air bearing-rotor system is shown in Figure 6. By comparing the rotor center trajectory under different external loads in Figure 6, it can be found that as the load increases, the size of the semi-major axis of the elliptical motion trajectory of the rotor gradually decreases, but the basic motion trajectory is the same as the characteristic vibration in Figure 5. Figure 6b,d,f,h also confirms the gradual decrease of the peak power spectral density of the half-speed whirl in the *x*-direction. The existence of the load increases the eccentricity of the hybrid air bearing. At large eccentricity, the damping of the system is enhanced, which can effectively limit the amplitude of the rotor movement.

Another typical non-linear dynamic behavior occurs when the rotating speed is 50,000 r/min, the system external load (vertical direction) is 30 N, the rotor mass is 90 g, and the unbalanced mass is 0.1 g, as depicted in Figure 7. Under this condition, the vibration amplitude and phase angle are both small. However, compared to Figure 5a, the increment of the unbalanced mass causes the rotor’s trajectory to be disturbed by the centrifugal force, which induces a hexagonal shape of rotor trajectory, as shown in Figure 7a. The peak of the power spectrum in the *x*-direction is still half-speed whirl motion in a system, as shown in Figure 7d. However, near the peak of half-speed frequency, another peak vibration frequency is caused by the unbalanced mass. The characteristic trajectory is formed by the centrifugal force together with the non-linear hybrid air bearing force.

The influences of different unbalanced masses on the center trajectory and power spectral density of the rotor are demonstrated in Figure 8. Figure 8a shows that when the unbalanced mass is zero, under the high dynamic stiffness and dynamic damping in the system, the trajectory of the rotor converges to a point. With the gradual increase of unbalanced mass, the maximum amplitude and perturbation energy of the rotor continue to increase, as shown in Figure 8c–f. As the unbalanced mass further increases, the energy of unbalanced mass is equivalent to the energy of the half-speed whirl motion, and the rotation becomes a ∞ shape, as shown in Figure 8g–i. However, with the further increment of unbalanced mass, the amplitude of rotation and the peak power spectral density of the unbalanced mass in the *x*-direction no longer increase, and the vibration is maintained within 0.5 μm.

The complicated trajectory appears when the rotor mass is 90 g, the unbalanced mass is 0.1 g, the speed is 70,000 r/min and the system external load (vertical direction) is 30 N, as shown in Figure 9. The larger rotating speed expands the amplitude and phase angle of the rotor in the *x*-direction in the trajectory of the rotor. This is because as the speed increases, the aerodynamic effect increases the stiffness in the *x*-direction. In the power spectral density, there are many peaks at other high-frequency frequencies in addition to half-speed whirl frequency. This is because as the speed increases, the vibration caused by the unbalanced mass inertia increases, thus the vibration gradually extends from half-speed whirl vibration to the combined effect of half-speed whirl and unbalanced mass combined vibration. This phenomenon is consistent with the research in reference [19].

Figure 10 illustrates the non-linear dynamics of the hybrid air bearing-rotor system at different rotating speeds. When the speed is lower than 40,000 r/min, as the speed increases, the trajectory of the rotor center gradually expands from a point to an ellipse, as shown in Figure 10a. When the speed reaches 40,000 r/min and around 140,000 r/min (the critical speed of the critical system), the vibration amplitude are significantly increased, as shown in Figure 10e,i. As the rotation speed continues to increase, the centrifugal force of the unbalanced mass increases, and the rotor trajectory changes from an elliptical network to a multi-ellipse superposition. The power spectral density also shows more vibration energy caused by the unbalanced mass, which reflects the influence of unbalanced mass on the non-linear force of the system, especially near the critical speed of the critical system.

For different working conditions, the structural parameters of the spindle system have different effects on the rotor trajectory of the spindle, especially in ultra-high-speed working conditions. Therefore, this paper further analyzes the influence of air film thickness, orifice diameter, length/diameter ratio and air supply pressure on the rotor trajectory of the hybrid air bearing-rotor system at ultra-high speed condition. In addition, this paper investigated the influence of two dynamic unbalance levels on the rotor trajectory under ultra-high speed conditions. Therefore, working conditions correspond to:

(1) Rotor mass 90 g, external load 30 N, ultra-high speed (150,000 r/min), dynamic unbalance grade G1 (corresponding to unbalanced mass 0.1 g);

(2) Rotor mass 90 g, external load 30 N, ultra-high speed (150,000 r/min), dynamic unbalance grade G0.1 (corresponding to unbalance mass 0.005 g).

**Figure 11:**
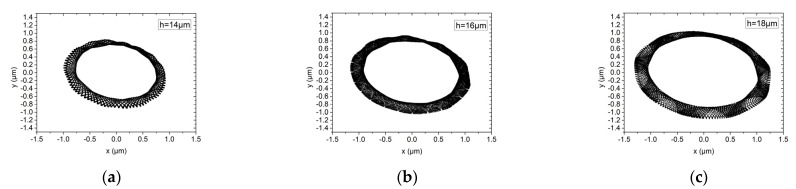
Rotor center trajectory under different gas film thickness under working condition 5 (high speed, dynamic unbalance G1), (**a**) rotor center trajectory (*h* = 14 μm), (**b**) rotor center trajectory (*h* = 16 μm), (**c**) rotor center trajectory (*h* = 18 μm).

**Figure 12:**
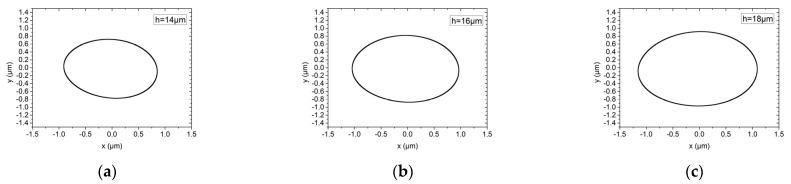
Rotor center trajectory under different gas film thickness under working condition 6 (high speed, dynamic unbalance G0.1). (**a**) Rotor center trajectory (*h* = 14 μm), (**b**) rotor center trajectory (*h* = 16 μm), (**c**) rotor center trajectory (*h* = 18 μm).

Figure 11 and Figure 12 show the influence of gas film thicknesses of 14 μm, 16 μm and 18 μm on the rotor trajectory of the hybrid air bearing-rotor system under working conditions (1) and (2), respectively. It can be seen that under rotating speed of up to 150,000 r/min, the maximum amplitude of the dynamic unbalance level G1 and G0.1 is about 0.8 μm under different gas film thicknesses. It can be seen that under the ultra-high speed condition, those with large unbalanced mass are belt-mounted elliptical trajectories, while those with small unbalanced mass are standard elliptical trajectories. This is because, at ultra-high rotating speed, the aerodynamic effect of the bearing system is significantly increased, and the peak energy density of the spindle system is mainly caused by the half-speed whirl motion of the hybrid air spindle. As the film thickness of the air film increases, the amplitude of the vibration of the spindle system will increase slightly.

**Figure 13:**
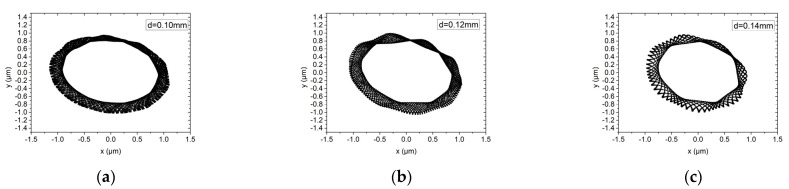
Rotor center trajectory under different orifice diameter under working condition 5 (high speed, dynamic unbalance G1). (**a**) Rotor center trajectory (*d* = 0.10 mm), (**b**) rotor center trajectory (*d* = 0.12 mm), (**c**) rotor center trajectory (*d* = 0.14 mm).

**Figure 14:**
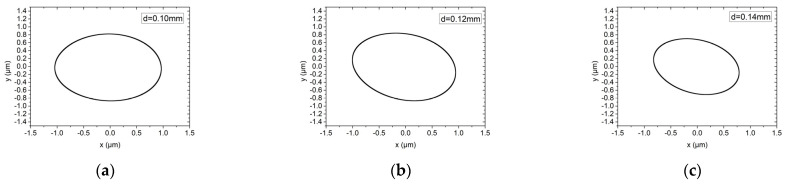
Rotor center trajectory under different orifice diameter under working condition 6 (high speed, dynamic unbalance G0.1). (**a**) Rotor center trajectory (*d* = 0.10 mm), (**b**) rotor center trajectory (*d* = 0.12 mm), (**c**) rotor center trajectory (*d* = 0.14 mm).

Figure 13 and Figure 14 show the influence of the orifice diameter on the rotor trajectory of the hybrid air bearing-rotor system under working conditions (1) and (2), respectively. The orifice diameters are 0.10 mm and 0.12 mm and 0.14 mm. Under ultra-high-speed working conditions, the system with larger orifice diameter could induce higher stiffness of the system and thus obtain smaller vibration amplitude. Similarly, different dynamic unbalance levels only affect the rotor trajectory of the hybrid air bearing-rotor system, and have little effect on the amplitude of the vibration amplitude of the rotor, which is different to the conclusion under the low speed condition. This is because the rotation of the spindle is no longer mainly restricted by the orifice under ultra-high-speed working conditions.

**Figure 15:**
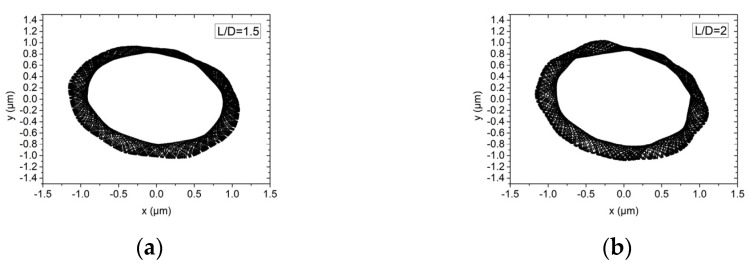
Rotor center trajectory under different L/D under working condition 5 (high speed, dynamic unbalance G1). (**a**) Rotor center trajectory (*L/D* = 1.5), (**b**) rotor center trajectory (*L/D* = 2).

**Figure 16:**
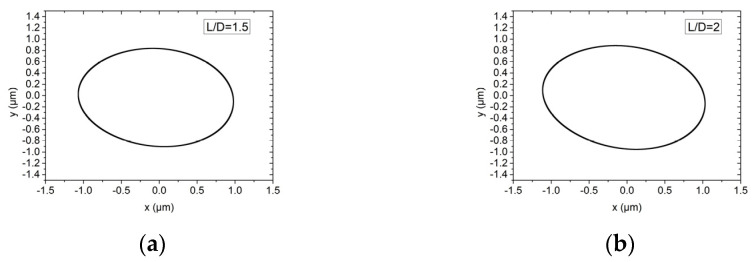
Rotor center trajectory under different L/D under working condition 6 (high speed, dynamic unbalance G0.1). (**a**) Rotor center trajectory (*L/D = 1.5*), (**b**) rotor center trajectory (*L/D = 2*).

The influence of the bearing with L/D of 1.5 and 2 on the rotor motion trajectory of the hybrid air bearing-rotor system is shown in Figure 15 and Figure 16. Air bearings that are too short will cause dynamic instability of the spindle, while bearings that are too long will reduce the load-bearing ratio and angular stiffness of the spindle. Therefore, in this study, L/D = 1.5 and L/D = 2 are selected for discussion. In ultra-high-speed working conditions, the vibration amplitudes of the dynamic balance levels G1 and G0.1 are both about 0.8 μm. As the L/D ratio increases, the rotation amplitude of the spindle system will increase slightly.

**Figure 17:**
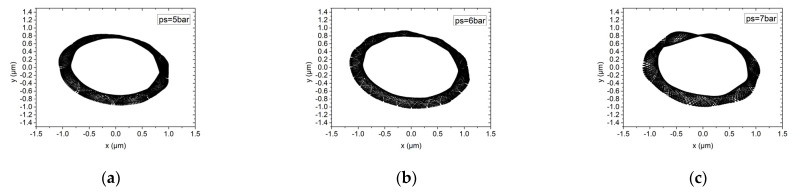
Rotor center trajectory under different supply pressure under working condition 5 (high speed, dynamic unbalance G1). (**a**) Rotor center trajectory (*ps* = 5 bar), (**b**) rotor center trajectory (*ps* = 6 bar), (**c**) rotor center trajectory (*ps* = 7 bar).

**Figure 18:**
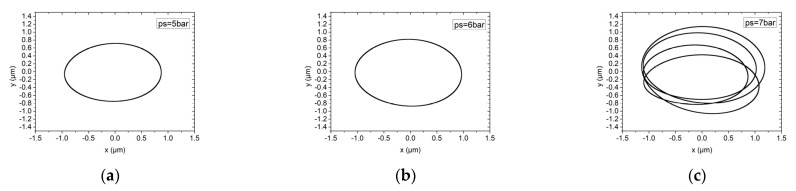
Rotor center trajectory under different supply pressure under working condition 6 (high speed, dynamic unbalance G0.1). (**a**) Rotor center trajectory(*ps* = 5 bar), (**b**) Rotor center trajectory (*ps* = 6 bar), (**c**) rotor center trajectory (*ps* = 7 bar).

Figure 17 and Figure 18 analyze the influence of the supply pressure on the rotor motion trajectory of the hybrid air bearing-rotor system. The supply pressure is 5 bar, 6 bar and 7 bar, respectively. Under ultra-high speed conditions, different air supply pressures have little effect on the hybrid pressure air bearing-rotor system with a dynamic balance level of G1, and the vibration amplitude of the rotor is about 0.8 μm. Similarly, in a system with a dynamic balance of G0.1, the influence of air supply pressure on the trajectory of the rotor center is also limited. However, in a system with an air supply pressure of 7 bar, the center trajectory of the rotor is characterized as a multi-elliptical T-shaped cross. This is because at this air supply pressure, the center of the rotor begins to significantly perturb at low frequencies. Therefore, at high speeds, the air supply pressure of the spindle should be kept below 6.5 bar.

## 4. Conclusions

In this paper, the non-linear analysis model of hybrid air bearing-rotor system in ultra-high speed condition is established based on solving unsteady Reynolds equations. The rotor trajectory is simulated together with the phase angle, time domain vibration and power spectral density for typical non-linear behaviors. Furthermore, the influences of rotor mass, external load, rotating speed and unbalanced mass on the nonlinear behavior of hybrid air bearings is investigated thoroughly. Finally, the influence of structure parameters on the trajectory of the hybrid air bearing-rotor systems under an ultra-high speed condition is studied in detail and the new phenomena of rotor trajectory under an ultra-high speed condition is illustrated. The following conclusions can be drawn:

(1) With the growth of the rotor mass, the amplitude of rotor trajectory obviously decreased and, meanwhile, the power spectral density also decreased to a low level. The increment of the external load only limited the amplitude of rotor trajectory but did not affect the typical non-linear behavior of the vibration.

(2) When unbalanced mass is small, the main cause of the non-linear behavior of the hybrid air bearing-rotor systems was the “half speed whirl”. During the increment of the unbalanced mass, rotor trajectory was combined with the centrifugal force caused by the half-speed whirl and the unbalanced mass, which was characterized as a ∞ shape.

(3) As the rotating speed reached 40,000 r/min and 140,000 r/min, which was the critical speed of the hybrid air bearing-rotor system, the power spectral density increased to a high level and the the rotor trajectory changed to a multi-ellipse superposition.

(4) Under ultra-high speed condition, the rotor trajectories of the hybrid air bearing-rotor systems with large unbalanced mass were belt-mounted elliptical trajectories, while those with small unbalanced mass were standard elliptical trajectories. By decreasing the film thickness and increasing the orifice diameter, the amplitude of the rotor trajectory could be restricted slightly under the ultra-high speed condition.

(5) Under the ultra-high speed condition, the L/D ratio and the supply pressure had little influence on the amplitude of the rotor trajectory of the hybrid air bearing-rotor system. However, a low-frequency vibration of the rotor was observed under the supply pressure of 7 bar, which was characterized as a multi-elliptical cross.

## Figures and Tables

**Table 1:** Bearing geometric parameters and operating parameters in this study.

Bearing Geometric Parameters and Operating Parameters	Value
Journal bearing diameter (*D*)	19.01 mm
Journal bearing length (*L*)	34.813 mm
Journal bearing clearance (*c*)	21.5 μm
Journal bearing Orifice diameter (*d*)	0.12 mm
Column number of feeding orifices	2
Number of orifice on each column	10
Axial distance between orifice and bearing end face (*L*_1_)	12.7 mm
Supplied pressure (*P_s_*)	6 bar

## Data Availability

Not applicable.

## Nomenclature

*C_d_*	Discharge coefficient
*d*	Orifice diameter (mm)
*e*	Eccentricity ratio
*f*	Approximate solution
*F_y_*	External force (N)
$\bar{h}$	Dimensionless air film thickness
*H*	Dimensionless groove depth (H = gd/c)
*l*	Length between orifice to edge
*L*	Bearing length(mm)
*m*	Rotor mass (kg)
*m_t_*	Mass flow rate (kg/s)
$m_{u}$	Unbalanced mass (kg)
*P*	Dimensionless air film pressure
$p_{a}$	Atmosphere pressure
$p_{s}$	Supply pressure
*u*, *v*	Velocity components in the x and z directions (m/s)
*Fx*, *Fy*	Moment in *x*- and *y*-direction(N)
$\bar{x}$, $\bar{z}$	Dimensionless coordinates
*x_Gq_*, y_Gq_	Rotor position
*λ*	Perturbation frequency ratio
*ψ*	Attitude angle
*θ*	Circumference angle
*η*	Air dynamic viscosity
$\xi_{i}$	Delta function
*ρ*	Air density (kg/m^3^)
$\rho_{a}$	Air density at atmosphere pressure (kg/m^3^)
*∧*	Bearing number
$\Lambda_{x}$, $\Lambda_{z}$	Dimensionless bearing numbers in the *x*- and *z*-direction
*κ*	Specific heat ratio of air
